# The patient perspective in research on major depression

**DOI:** 10.1186/1471-244X-11-89

**Published:** 2011-05-18

**Authors:** Pim Cuijpers

**Affiliations:** 1 Pim Cuijpers, professor of Clinical Psychology, EMGO Institute, VU University Amsterdam and VU University Medical Center, Department of Clinical Psychology Van der Boechorststraat 11081 BT Amsterdam The Netherlands

## Abstract

Although thousands of studies have examined the genetics, epidemiology, etiology, biology, treatment and prevention of major depressive disorder, we still lack very basic knowledge about what patients with depressive disorders need. Despite the thousands of studies that have been conducted on major depression and the hundreds of randomized trials that have examined the effects of treatments, many patients still do not know how to cope with the daily problems caused by depressive disorders. In this Commentary the need for more research on the perspectives of patients is described. This research should guide treatment studies as well as basic research much more than it currently does. This perpective is especially important to understand and solve the undertreatment of depression, one of the major problems in this area. Up to 50% of depressed patients do not seek treatment, resulting in huge avoidable disease burden and economic costs. In order to solve this problem we need a better understanding of the problems patients encounter in daily life, and what factors contribute to the reasons for seeking treatment or not. Research from the patients' perspective is also necessary to meet the currently unmet information needs of patients, including information about the nature and causes of depression, stigma, medication, treatment and coping with the daily problems of having depression.

## Commentary: The patient perspective in research on major depression

In the past decades, thousands of studies have examined the genetics, epidemiology, etiology, biology, treatment and prevention of major depressive disorder. This huge body of research has resulted in extensive knowledge about major depressive disorders, what it is, who are at increased risk of getting it, which processes lead to it, how it can be treated, and even in some cases be prevented. Not all research questions have been answered and we will indoubtedly need several more decades to fully understand the phenomenon of depression. But we currently do have considerably more than a basic understanding of what it is and how it can be treated.

Despite these major advances of science in this field, very basic knowledge about what patients with depressive disorders need is still lacking. That is probably the most important message from the paper by Barney, Griffiths and Banfield in *BMC Psychiatry *about information needs of people with depression [1]. They show that despite the hundreds of randomized trials examining the effects of pharmacotherapy and psychotherapies for depression, many patients still do not know how to cope with the daily problems caused by depressive disorders. Despite the availability of effective treatments, many patients still doubt whether these treatments actually work, are still afraid for negative responses from their environment and do not know how to deal with these. And despite the sophisticated models that have been developed to deliver effective treatments, many patients still do not know where to get that treatment.

Researchers need the perspectives of patients and the problems they experience in daily life more than ever. It is not enough to show in a randomized trial that a medicine is effective for the average patient, to show that a specific psychological treatment is also effective in postpartum depression or for older adults, to show that a preventive intervention reduces the incidence of new cases of depressive disorders. We need to get a better understanding of what it means to live with a depressive disorder, what problems a patient encounters when he or she goes to work, communicates with friends and relatives, when he or she can not sleep at night, or suffers from severe side effects of medications. Only these issues can help us understand which treatments are needed and how they can reduce the enormous disease burden of major depression. Such questions should guide researchers much more than currently occurs. At this moment very little research focuses on the perspectives of patients. A search in PubMed with the Mesh heading "major depression" resulted in 67,805 hits. When we limit this to "Patient Advocacy" only 54 remained (accessed on April 18, 2011). That is 0.07%. And if we limit it to "Patient Rights" we find 387 hits (0.6%).

One of the major problems in the treatment of depressive disorders is that up to 50% of depressed patients do not seek treatment, resulting in huge avoidable disease burden and economic costs [2]. Although the outcome of untreated depression is not necessarily worse than the outcome in patients receiving treatment [3], many of the untreated patients do have a clear need for treatment [4]. This research question can only be answered through examining the perspectives of patients in their illness and how to cope with it. There are many reasons for not seeking treatment including patient and provider factors. Provider factors include among others the stigma still associated with depression, poor professional education about depression, limited training in interpersonal skills, and inadequate time to evaluate and treat depression [5]. There are also several reasons why patients themselves do not seek treatment when this would be appropriate, including the stigma, financial reasons, the idea that treatments do not work, and not knowing where to get help [6]. In order to solve the problem of untreated depression we need a better understanding of the problems patients encounter in daily life, and what factors contribute to the reasons for seeking treatment or not. Although some research has been conducted in this area, this phenomenon is still not well understood, and more importantly, what can be done about it. We need more research from the patients' perspective to understand and solve this problem.

The study by Barney, Griffiths and Banfield also points at another major problem from the patients' perspective: the substantial unmet information needs of patients, which includes information about the nature and causes of depression, stigma, medication, treatment and coping with the daily problems of having depression. Much of this information is widely available on the Internet. It is not only the availability of this information, however, that needs our attention. It is also the way in which it is presented to patients. Doctors and therapists should be the first to give adequate information to patients, although the dialogue between patients and doctors is often suboptimal in this respect [5]. Patients can also find the needed information themselves on the Internet and in self-help books. But peer support will also remain one of the important sources of information. Patients prefer to hear good information from other people who have gone through the same problems, and learn from each others' experiences.

It is not a coincidence that the study by Barney, Griffiths and Banfield uses an Internet-based support group for their research. The Internet not only offers excellent opportunities to organise mutual support groups, it also has great potential to examine mutual support processes. Large numbers of patients engage every day in Internet-based forums with other patients [7]. This is an excellent way to organise mutual support for patients, that empowers patients and may have some beneficial effects on users [8]. But it also is a huge resource for examining these processes for researchers. It results in enormous amounts of texts that can be analysed to get a better understanding of what patients need.

We must conclude that despite decades of research on depressive disorders, we still do not know very well what having a depression means for patients, what they need from treatments and how their problems can be solved. We need this knowledge, however, to get a better understanding of the phenomenon of depression, its causes and treatment. What are we waiting for?

## Competing interests

The author declares that they have no competing interests.

## Pre-publication history

The pre-publication history for this paper can be accessed here:

http://www.biomedcentral.com/1471-244X/11/89/prepub
